# Valvular and ascending aortic hemodynamics of the On-X aortic valved conduit by same-day echocardiography and 4D flow MRI

**DOI:** 10.3389/fcvm.2023.1256420

**Published:** 2023-11-14

**Authors:** Jeesoo Lee, Hyungkyu Huh, Michael B. Scott, Mohammed S. M. Elbaz, Jyothy J. Puthumana, Patrick McCarthy, S. Christopher Malaisrie, Michael Markl, James D. Thomas, Alex J. Barker

**Affiliations:** ^1^ Department of Radiology, Feinberg School of Medicine, Northwestern University, Chicago, IL, United States; ^2^ Medical Device Development Center, Daegu-Gyeongbuk Medical Innovation Foundation, Daegu, Republic of Korea; ^3^ Department of Biomedical Engineering, McCormick School of Engineering, Northwestern University, Evanston, IL, United States; ^4^ Department of Cardiology, Feinberg School of Medicine, Northwestern University, Chicago, IL, United States; ^5^ Division of Cardiac Surgery, Feinberg School of Medicine, Northwestern University, Chicago, IL, United States; ^6^ Department of Radiology, University of Colorado Anschutz Medical Campus, Aurora, CO, United States

**Keywords:** bileaflet mechanical aortic valved conduit, valvular hemodynamics, ascending aortic hemodynamics, 4D flow MRI, echocardiography

## Abstract

This study aims to assess whether the On-X aortic valved conduit better restores normal valvular and ascending aortic hemodynamics than other commonly used bileaflet mechanical valved conduit prostheses from St. Jude Medical and Carbomedics by using same-day transthoracic echocardiography (TTE) and 4D flow magnetic resonance imaging (MRI) examinations. TTE and 4D flow MRI were performed back-to-back in 10 patients with On-X, six patients with St. Jude (two) and Carbomedics (four) prostheses, and 36 healthy volunteers. TTE evaluated valvular hemodynamic parameters: transvalvular peak velocity (TPV), mean and peak transvalvular pressure gradient (TPG), and effective orifice area (EOA). 4D flow MRI evaluated the peak systolic 3D viscous energy loss rate (VELR) density and mean vorticity magnitude in the ascending aorta (AAo). While higher TPV and mean and peak TPG were recorded in all patients compared to healthy subjects, the values in On-X patients were closer to those in healthy subjects (TPV 1.9 ± 0.3 vs. 2.2 ± 0.3 vs. 1.2 ± 0.2 m/s, mean TPG 7.4 ± 1.9 vs. 9.2 ± 2.3 vs. 3.1 ± 0.9 mmHg, peak TPG 15.3 ± 5.2 vs. 18.9 ± 5.2 vs. 6.1 ± 1.8 mmHg, *p* < 0.001). Likewise, while higher VELR density and mean vorticity magnitude were recorded in all patients than in healthy subjects, the values in On-X patients were closer to those in healthy subjects (VELR: 50.6 ± 20.1 vs. 89.8 ± 35.2 vs. 21.4 ± 9.2 W/m^3^, *p* < 0.001) and vorticity (147.6 ± 30.0 vs. 191.2 ± 26.0 vs. 84.6 ± 20.5 s-1, *p* < 0.001). This study demonstrates that the On-X aortic valved conduit may produce less aberrant hemodynamics in the AAo while maintaining similar valvular hemodynamics to St. Jude Medical and Carbomedics alternatives.

## 1. Introduction

Bileaflet mechanical aortic valves are commonly recommended for aortic valve replacement (AVR) in patients with a longer life expectancy [age ≤50 (1) or 60 years (2)] due to their long-lasting structural durability over bioprosthetic valves (1–3). However, mechanical valves are more prone to thrombus formation, which requires patients to undergo a lifelong anticoagulation therapy, subsequently increasing the risk of bleeding events compared to bioprosthetic valves (35.1% vs. 23.3% in patients 45–54 years of age) (3–6). The On-X aortic valve (On-X Life Technologies, Kennesaw, GA) is currently the only mechanical heart valve approved by the FDA for a lower international normalized ratio target of 1.5–2.0 (cf., standard range = 2.0–3.0) (7). Further, improved valvular hemodynamics has been reported for the On-X valve compared to other bileaflet mechanical valves, as marked by reduced transvalvular peak velocity (TPV), transvalvular pressure gradient (TPG), and increased effective orifice area (EOA) (8–10). However, the hemodynamic impact of AVR with a valved conduit is unclear since the changes in Windkessel performance of a stiff conduit vs. native elastic aortic tissue may impact cardiac afterload and downstream hemodynamics (11).

Time-resolved three-dimensional (3D) phase-contrast magnetic resonance imaging (MRI), commonly referred to as 4D flow MRI (12), is capable of visualizing 3D aortic flow behavior and has been utilized to assess the impact of different aortic valve prostheses on aortic hemodynamics (13–15). For example, von Knobelsdorff-Brenkenhoff et al. observed aberrant vortical and helical ascending aorta (AAo) flow patterns in AVR patients with autograft, mechanical, and bioprosthetic valves, including those with bileaflet mechanical valves (14). In particular to the On-X valved conduit, Keller et al. reported similar helical and vortical flow patterns in AAo between patients and healthy controls (15). However, prior studies have only evaluated qualitative 3D aortic flow patterns using semi-quantitative scoring, limiting the data reproducibility. Further, valvular hemodynamics was not evaluated due to susceptibility image artifacts induced by the valve.

This pilot study conducted a comprehensive and quantitative evaluation of valvular and ascending aorta (AAo) hemodynamics of the On-X aortic valved conduit in comparison to healthy subjects and other commonly used prostheses, namely, St. Jude Medical (SJM) and Carbomedics (CM), using same-day transthoracic echocardiography (TTE) and 4D flow MRI examinations. Valvular hemodynamics was characterized by clinical standard TTE measures (TPV, mean and peak TPG, and EOA). AAo hemodynamics was characterized by computing viscous energy loss and vorticity at peak systole. This study aims to examine whether the On-X aortic valved conduit restores normal valvular and AAo hemodynamics better than the other prostheses.

## 2. Materials and methods

### 2.1. Study cohort

This study was approved by the Institutional Review Board, and written informed consent was obtained from all subjects. Inclusion criteria were adult patients (age 18–89 years) who underwent Bentall procedure on or after 1 January 2001 using the following bileaflet mechanical aortic valved conduit prostheses: On-X Ascending Aortic Prosthesis with Vascutek Gelweave Valsalva Graft (On-X), SJM Masters HP Valved Graft with Gelweave Valsalva Technology, or CM Carbo-seal Valsalva. Seventeen patients were prospectively enrolled between October 2017 and June 2020 and divided into two groups: (1) On-X (*n* = 11) and (2) SJM (*n* = 2) or CM (*n* = 4). Thirty-six healthy subjects with a tricuspid aortic valve who underwent same-day TTE and 4D flow MRI examinations for another study were retrospectively recruited.

### 2.2. Echocardiography

A standard-of-care TTE was performed using a Vivid E95 echocardiography scanner (General Electric Healthcare, Waukesha, WI, USA). TPV, mean and peak TPG, EOA, and EOA index (EOAi) were evaluated based on continuous-wave Doppler and 2D color Doppler echocardiography following the American Society of Echocardiography guidelines (16). To briefly explain how EOA was measured, first, the left ventricle stroke volume at the left ventricle outflow tract was obtained by multiplying the velocity-time-integral of forward flow (toward the aortic valve) by the cross-sectional area, which are measured using pulsed-wave Doppler and parasternal long-axis B-mode echocardiography, respectively. Given the principle of mass conservation, the stroke volume at the left ventricle outflow tract should be equal to that at the aortic valve. The stroke volume at the left ventricle outflow tract was divided by the velocity-time-integral of the forward transaortic valvular flow, which is measured using aortic valve continuous-wave Doppler echocardiography, giving EOA. In addition, a discharge coefficient, defined as EOA normalized by a geometrical orifice area (i.e., the area when a valve is fully opened), was introduced to account for valve label size differences in patients. The geometrical orifice area was obtained from the valve specification sheet provided by the respective valve manufacturers. The discharge coefficient indicates the ability of the valve to distribute blood flow effectively across the valve.

### 2.3. 4D flow MRI

**Figure 1:**
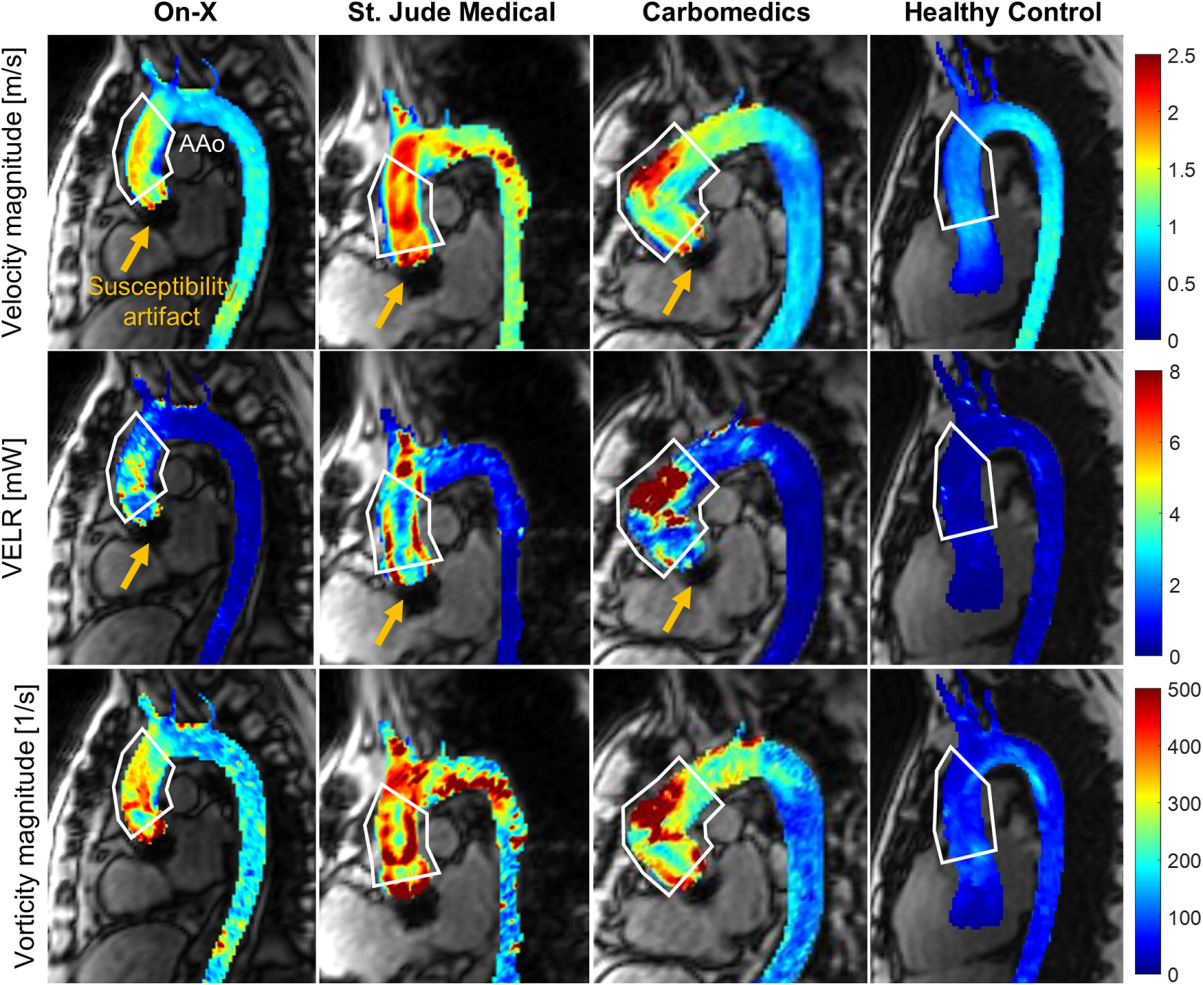
Example images showing the peak systolic aortic flow field quantified by 4D flow MRI in patients with the on-X aortic valve conduit (column 1), St. Jude Medical (column 2), Carbomedics (column 3), and healthy subjects (column 4). The velocity magnitude (row 1) and vorticity magnitude (row 3) are shown as the maximum intensity projection, and VELR (row 2) indicates the viscous energy loss rate. The white polygons indicate the ascending aortic region of interest. Note that the yellow arrows indicate the region of susceptibility artifact due to the presence of a mechanical aortic valve. The temporal evolution of 3D aortic flow in the subjects presented in this figure is visualized using pathlines and is available as Supplementary Videos.

All MRI examinations were conducted on a 1.5-T system (Aera, Siemens Healthineers, Erlangen, Germany). A retrospective electrocardiogram and respiratory-gated, free-breathing 4D flow MRI examination was performed with the following parameters; echo time = 2.1–2.3 ms, repetition time = 4.8–5.1 ms, flip angle = 7°, temporal resolution = 38.8–40.6 ms, bandwidth = 455 Hz/pixel, field of view = 285–345 × 380–460 × 72–96 mm^3^, matrix size = 120 × 160 × 30–32, voxel size = 3.4–4.1 × 2.4–2.9 × 2.4–3 mm^3^, and encoding velocity = 150–275 cm/s. All 4D flow MRI datasets were corrected for background phase offset and velocity aliasing using previously described methods (17). The entire thoracic aorta was segmented using a deep learning technique described previously (18). The AAo was then defined manually from the sinotubular junction to the first brachiocephalic branch (Figure 1, white line). The peak systole time point is defined as the frame at which the average velocity magnitude of the blood in the aorta is at its maximum. At peak systole, the viscous energy loss rate (VELR) and a vorticity vector were computed per voxel, providing a 3D map, as shown in Figure 1, columns 2 and 3, respectively. VELR represents the rate of flow mechanical energy loss due to friction between two adjacent fluid layers moving at a different velocity (i.e., fluid shear) (19). Vorticity represents the angular velocity vector of a fluid element under rotation. The magnitude of vorticity increases in regions of large velocity gradient, which indicates a spatially non-uniform flow field (i.e., less organized or chaotic). Mathematical definitions of VELR and vorticity are provided in the Supplementary Material. Total VELR, which is the sum of all voxelwise VELR values in the AAo, VELR density (total VELR divided by AAo volume), and mean vorticity, which is the sum of vorticity magnitude in the AAo divided by AAo volume, were computed. In addition, time-resolved 3D flow patterns in the aorta were visualized by generating 3D flow pathlines using Ensight (2022 R1, Ansys, Canonsburg, PA, USA). Each pathline represents a trajectory of a zero-mass particle seeded in the aorta segmentation over a 40 ms time window. A total of 400 zero-mass particles were continuously seeded in the entire aorta with a 40 ms interval. The aortic flow pathlines of the subjects presented in Figure 1 are included as Supplementary Videos S1–S4.

### 2.4. Statistical analysis

Average statistics are reported using the mean and standard deviation or median and interquartile range depending on data normality determined by the Lilliefors test. One-way analysis of variance or the Kruskal–Wallis test was used for evaluating differences between all three groups (On-X vs. SJM/CM vs. healthy controls) with a significance level of *p* < 0.05. Pairwise differences between the two groups were tested using a two-tailed unpaired *t*-test or the Wilcoxon rank-sum test with a significance level of *p* < 0.017 adjusted using the Bonferroni correction.

## 3. Results

### 3.1. Study cohort characteristics

One On-X patient could not complete an MRI exam due to claustrophobia and thus was excluded from the study. Baseline characteristics of the patients (*n* = 10 On-X, *n* = 6 SJM/CM) and healthy subjects (*n* = 36) are listed in Table 1. The age and body surface area were not statistically different between the three groups. The left ventricle end-diastolic volume (131 ± 25 vs. 161 ± 56 ml) and end-systolic volume (55 ± 7 vs. 69 ± 22 ml) were not different between the On-X and SJM/CM patients but were larger than those of healthy subjects (end-diastolic volume 97 ± 33 ml and end-systolic volume 36 ± 16 ml). The left ventricle ejection fraction in both patient groups was lower than in healthy subjects but within the normal range (52%–72%). At the time of imaging, all patients had undergone surgery more than 1 year ago, but On-X patients had a shorter postoperative period than SJM/CM patients [1,561 (1,198–1,629) days vs. 2,283 (1,797–2,747) days].

**Table 1:** Study cohort characteristics.

Characteristics	On-X (*n* = 10)	SJM/CM (*n* = 6)	Healthy control (*n* = 36)	*p*-value On-X vs. SJM/CM	*p*-value On-X vs. healthy	*p*-value SJM/CM vs. healthy	*p*-value ANOVA
Gender	M 9/F 1	M 4/F 2	M 15/F 21				
Age	49 ± 14	45 ± 12	52 ± 10	—	—	—	—
BSA (m^2^)	2.1 ± 0.2	2.2 ± 0.3	1.9 ± 0.2	—	0.023	0.03	—
End-diastolic LV volume (ml)*^,^**	131 ± 25	161 ± 56	97 ± 33	—	0.004	<0.001	<0.001
End-systolic LV volume (ml)*^,^**	55 ± 7	69 ± 22	36 ± 16	—	<0.001	0.001	<0.001
EF (%)*^,^**	57 ± 6	57 ± 5	64 ± 10	—	0.002	0.003	<0.001
Graft diameter (mm)	26 (26–26)	30 (26–30)	n.a.	—	—	—	—
Valve label size (mm)	25 (25–25)	28 (25–31)	n.a.	—	—	—	—
Geometric orifice area (cm^2^)	3.73 (3.73–3.73)	4.14 (3.16–5.18)	n.a.	—	—	—	—
Days after surgery***	1,561 (1,198–1,629)	2,283 (1,797–2,747)	n.a.	0.016	—	—	—

LV, left ventricle; BSA, body surface area; EF, ejection fraction.

The listed *p*-values are three-group comparison results. Significant differences between the two groups are denoted as follows: *On-X vs. healthy controls; **SJM/CM vs. healthy controls; ***On-X vs. SJM/CM.

### 3.2. Hemodynamic assessment

Hemodynamic assessment results are listed in Table 2. Valvular hemodynamic assessments showed that On-X patients had elevated TPV (1.9 ± 0.3 vs. 1.2 ± 0.2 m/s, *p* < 0.001), mean TPG (7.4 ± 1.9 vs. 3.1 ± 0.9 mmHg, *p* < 0.001), and peak TPG (15.3 ± 5.2 vs. 6.1 ± 1.8 mmHg, *p* < 0.001) compared to healthy subjects, while SJM/CM patients had even higher values (TPV 2.2 ± 0.3 m/s, mean TPG 9.2 ± 1.9 mmHg and peak TPG 18.9 ± 5.2 mmHg, all *p*’s < 0.001). However, the difference between On-X and SJM/CM patients was not statistically significant. The EOA and EOA indexes in On-X and SJM/CM patients were not different from each other or healthy subjects. The discharge coefficient was also not different between the On-X and SJM/CM patients.

**Table 2:** Hemodynamic assessment.

Measurements	On-X (*n* = 10)	SJM/CM (*n* = 6)	Healthy controls (*n* = 36)	*p*-value On-X vs. SJM/CM	*p*-value On-X vs. healthy	*p*-value SJM/CM vs. healthy	*p*-value ANOVA
Valvular hemodynamics							
TPV (m/s)*^,^**	1.9 ± 0.3	2.2 ± 0.3	1.2 ± 0.2	—	<0.001	<0.001	<0.001
Mean TPG (mmHg)*^,^**	7.4 ± 1.9	9.2 ± 2.3	3.1 ± 0.9	—	<0.001	<0.001	<0.001
Peak TPG (mmHg)*^,^**	15.3 ± 5.2	18.9 ± 5.2	6.1 ± 1.8	—	<0.001	<0.001	<0.001
EOA (cm^2^)	2.51 ± 0.58	2.53 ± 0.64	2.3(2.1–2.8)	—	—	—	—
EOAi (cm^2^/m^2^)	1.21 ± 0.34	1.18 ± 0.30	1.3 ± 0.30	—	—	—	—
Discharge coefficient	0.69 ± 0.14	0.65 ± 0.20	—	—	—	—	—
Ascending aortic hemodynamics							
Total VELR (mW)*^,^**^,^***	3.1 ± 1.0	6.0 ± 4.1	1.6 ± 0.7	0.009	<0.001	<0.001	<0.001
VELR density (W/m^3^)*^,^**^,^***	50.6 ± 20.1	89.8 ± 35.2	21.4 ± 9.2	0.013	<0.001	<0.001	<0.001
Mean vorticity (s^−1^)*^,^**^,^***	147.6 ± 30.0	191.2 ± 26.0	84.6 ± 20.5	0.011	<0.001	<0.001	<0.001

TPV, transvalular peak velocity; TPG, transvalvular pressure gradient; EOA, effective orifice area, EOAi, effective orifice area index; VELR, viscous energy loss rate.

The listed *p*-values are three-group comparison results. Significant differences between the two groups are denoted as follows: *On-X vs. healthy controls; **SJM/CM vs. healthy controls; ***On-X vs. SJM/CM.

**Figure 2:**
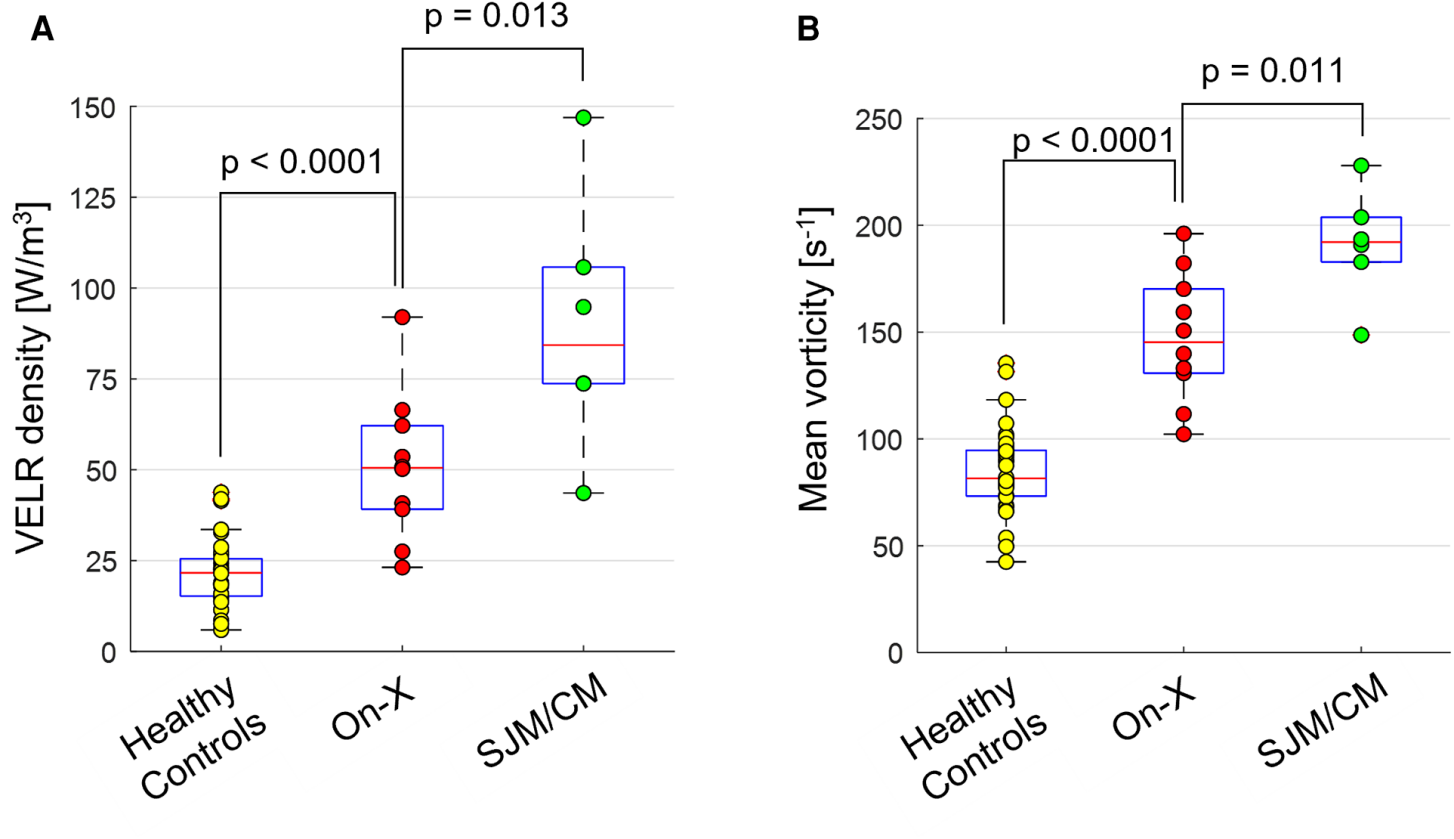
Boxplots showing the distribution of (**A**) viscous energy loss rate (VELR) density and (**B**) mean vorticity magnitude within each group.

Ascending aortic hemodynamic assessments demonstrated similar trends to valvular hemodynamics. Compared to healthy subjects, On-X patients had significantly higher total VELR (3.1 ± 1.0 vs. 1.6 ± 0.7 mW, *p* < 0.001), VELR density (50.6 ± 20.1 vs. 89.8 ± 35.2 W/m^3^, *p* < 0.001), and mean vorticity (147.6 ± 30.0 vs. 84.6 ± 20.5 s^−1^, *p* < 0.001), while SJM/CM patients had even higher values (total VELR 6.0 ± 4.1 mW, VELR density 89.8 ± 35.2 W/m^3^, and mean vorticity 191.2 ± 26.0 s^−1^, all *p*’s < 0.001 vs. healthy subjects). The differences in VELR density and mean vorticity between On-X and SJM/CM patients were statistically significant (*p* < 0.017), as shown in Figure 2.

## 4. Discussion

This is a pilot study that utilized same-day TTE and 4D flow MRI examinations for a comprehensive assessment of valvular and AAo hemodynamics of the On-X aortic valved conduit compared with similar aortic valved conduits from SJM and CM and with healthy subjects. Our study suggests that while there may be no significant improvements in valvular hemodynamics, the On-X prosthesis may produce less aberrant flow in the AAo as indicated by lower viscous energy loss and vorticity magnitude that are closer to those of healthy subjects compared to the SJM and CM alternatives.

Elevated VELR and vorticity have been associated with aberrant ascending aortic flow patterns such as helical flow, vortical flow, and flow jets, which are frequently observed in patients with aortic valve disease, including aortic stenosis and bicuspid aortic valve (20, 21). Previous studies have reported abnormal helical and vortical flow patterns in patients with various types of AVR, including SJM and CM bileaflet mechanical aortic valves (13, 14). In contrast, patients with the On-X valved conduit showed flow patterns similar to healthy volunteers (15). Our study found lower VELR and vorticity in patients with the On-X valved conduit than those with alternative SJM and CM bileaflet mechanical aortic valved conduits, but the values were still higher than those of healthy subjects who demonstrated the most aligned ascending aortic flow patterns compared to patients based on qualitative assessment (Supplementary Video S1–S4). Considering our results in conjunction with previous studies, the On-X aortic valved conduit may perform better in mitigating aberrant flow patterns in the aorta compared to other SJM and CM alternatives but may not completely restore normal flow patterns.

Since all three prostheses share the same Gelweave graft with preserved sinuses of Valsalva, the valve design is likely the major factor responsible for hemodynamic differences between the two patient groups. The On-X valve has a flared inlet, while the other two have a straight inlet. This tapered inlet may have facilitated the smooth entry of blood flow from the left ventricle outflow tract, promoting organized flow stream and thus preventing complex flow formation in the aorta (22). Another design feature of the On-X valve is the 90° valve leaflet opening angle, which is higher than those of SJM (85°) and CM (78°). The ability to fully open may be more beneficial in terms of forming coherent centrally aligned flows, while valves with an opening angle of less than 90° create flows directed toward the aortic wall that may initiate secondary helical and vortical flow patterns, as observed in a previous study (14). In addition to the differences in valve design, individual variation in the graft curvature may also alter downstream flow formation as it affects the impact angle of transaortic valvular flow on the graft wall. However, within our limited patient cohort, there was no noticeable relationship between the graft curvature and VELR or mean vorticity magnitude among patients with the same prosthesis (Supplementary Figure S1 and S2).

Given that peak and mean TPG were not significantly different between the two patient groups, the lower VELR density in the On-X patients may indicate that the On-X prosthesis provides a more energy-efficient AAo flow since viscous energy loss is one of the irreversible pressure energy losses that occur in the downstream of the valve. A reduction in irreversible energy loss leads to higher pressure recovery (23, 24) in the aorta; thus reducing the work the left ventricle must produce to push blood into the aorta. It is also worth noting that VELR in the AAo was analyzable and not limited by the presence of a mechanical aortic valve. The size of the magnetic susceptibility artifact (i.e., signal void region), as depicted in Figure 1, measured approximately 1.5 cm along the aorta. Velocities in the AAo, defined as the sinotubular junction to the brachiocephalic artery, remained unaffected by the artifact. Another significant source of irreversible energy loss is turbulent dissipation, which requires high temporal resolution measurement of random velocity fluctuations (i.e., turbulence). The 4D flow MRI technique used in this study does not have sufficient temporal resolution for direct turbulence measurement and only acquires blood velocities averaged over multiple heartbeats. A special 4D flow MRI sequence such as ICOSA6 (25) designed to quantify average turbulent kinetic energy may be utilized; however, it requires further validation in *in vivo* pulsatile flow scenarios.

While a similar to or higher EOA has been reported for the On-X valve than the SJM or CM valve for the same valve label size (8, 10, 26), our results showed no significant difference between On-X and SJM/CM patients. However, the size of the implanted valve widely varied in the SJM/CM patients (ranging from 23 to 31 mm), while the On-X patients only had two sizes: 23 or 25 mm. To eliminate the confounding effects of size heterogeneity, we computed the discharge coefficient, which is the ratio of EOA to the geometrical orifice area. The discharge coefficient provides an indication of the valve design's effectiveness in constricting blood flow. Our results showed that the discharge coefficient was similar between the two patient groups (0.69 ± 0.14 vs. 0.65 ± 0.20, *P* = 0.65). This suggests that the unique design features of the On-X valve did not provide any significant valvular hemodynamic advantage over the other two valves. A previous *in vitro* study observed that the On-X valve leaflets experienced fluctuation during systole, which may affect the valve's ability to remain fully open, while the SJM valve leaflets remained stable (27). This could be due to the leaflets opening at 90°, which results in the absence of supporting force compared to leaflets that fully open at <90°, which experience a constant pressure force from the incoming flow. This leaflet instability may cause flow separation at the valve, blocking blood flow through the valve and reducing EOA.

There are several limitations in this study. First, the small patient sample size critically limits the statistical power of the study. Second, the comparison group consisted of two different mechanical valves, which may introduce confounding variables that could affect the results. However, the patient cohort in this study presented a significant recruitment challenge due to the low prevalence of mechanical AVR, which is only performed in about 10% of AVR patients, and the even lower frequency of Bentall procedures. Third, the valve label size and graft size were not controlled between the On-X and SJM/CM groups, where the sizes varied more widely in SJM/CM patients. We compensated for these size discrepancies by introducing the discharge coefficient and normalizing total VELR and vorticity by the AAo volume. Finally, downstream flow turbulence was not examined due to technical limitations, and thus, the downstream energetic efficiency was not fully investigated. A carefully designed *in vitro* experiment with a high-resolution velocity imaging technique such as particle image velocimetry may be a valuable approach to analyzing detailed turbulent flow structures and identifying sources of turbulence. Nonetheless, this study provides a valuable three-dimensional assessment of the hemodynamic performance of the On-X aortic valved conduit compared to other mechanical valves.

## Data Availability

The raw data supporting the conclusions of this article will be made available by the authors per request, without undue reservation.

## Supplementary material

Supplementary Figure S1 Maximum intensity projection of the viscous energy loss rate (VELR) at peak systole in all patients. The diameter of the implanted graft is indicated below each image.

Supplementary Figure S2 Maximum intensity projection of vorticity magnitude at peak systole in all patients. The diameter of the implanted graft is indicated below each image.

Supplementary Video S1 Time-resolved 3D aortic blood flow pattern in a patient with On-X prosthesis.

Supplementary Video S2 Time-resolved 3D aortic blood flow pattern in a patient with St. Jude Medical Masters prosthesis.

Supplementary Video S3 Time-resolved 3D aortic blood flow pattern in a patient with Carbomedic prosthesis.

Supplementary Video S4 Time-resolved 3D aortic blood flow pattern in a healthy volunteer.
