# Supplementary figures and images for: Valvular and ascending aortic hemodynamics of the On-X aortic valved conduit by same-day echocardiography and 4D flow MRI
Source: Front Cardiovasc Med. 2023 Nov 14;10:1256420. doi: 10.3389/fcvm.2023.1256420 (PMC10682731; doi:10.3389/fcvm.2023.1256420)

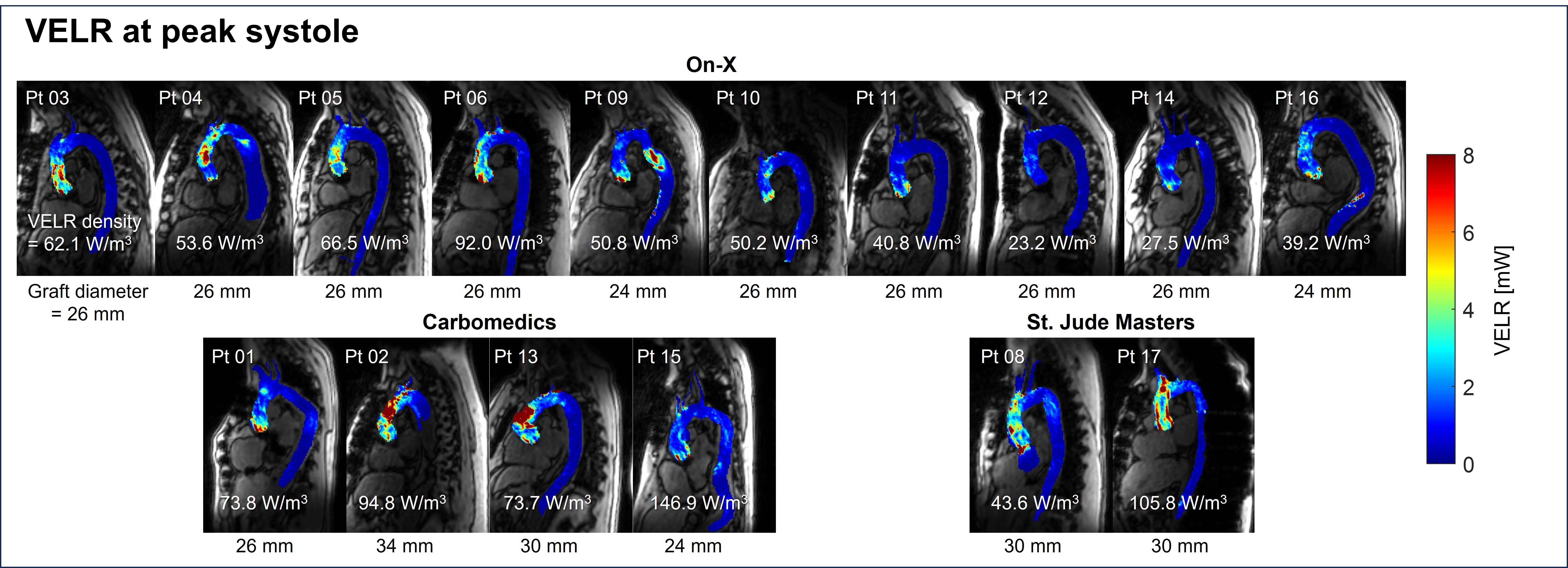

Supplement: Supplementary Figure S1 — Maximum intensity projection of the viscous energy loss rate (VELR) at peak systole in all patients. The diameter of the implanted graft is indicated below each image. [file Image1.jpeg]

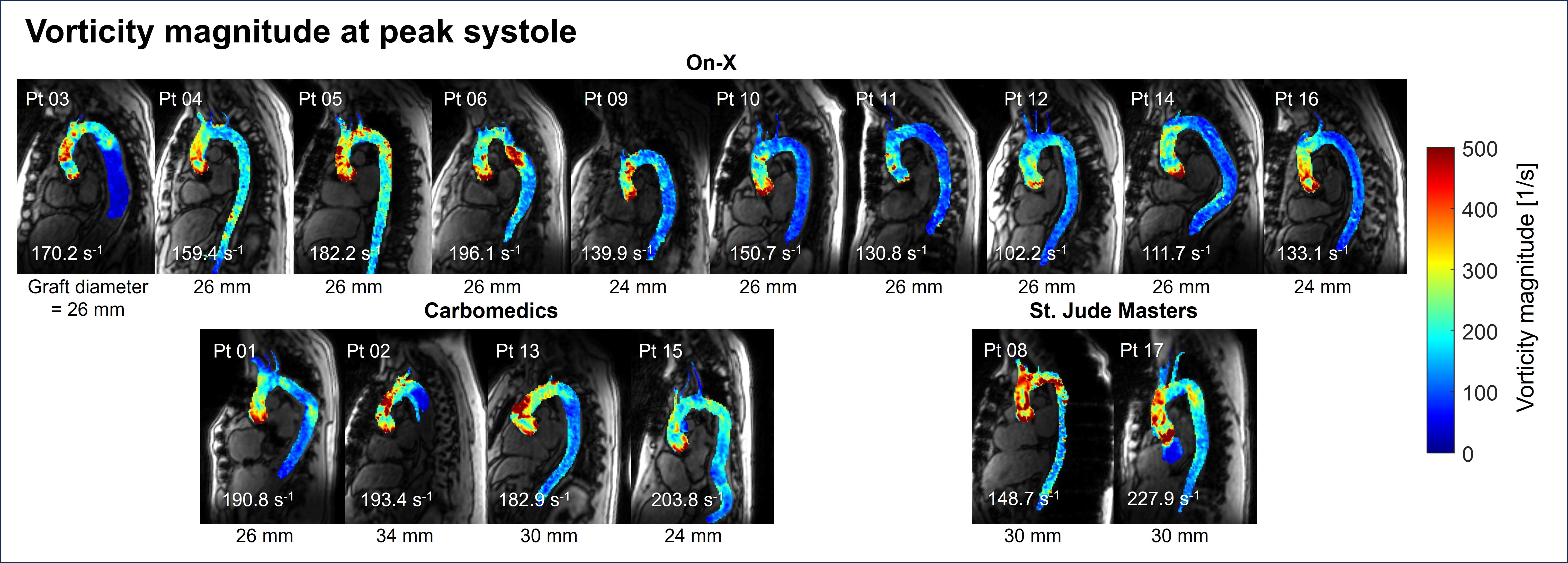

Supplement: Supplementary Figure S2 — Maximum intensity projection of vorticity magnitude at peak systole in all patients. The diameter of the implanted graft is indicated below each image. [file Image2.jpeg]
