# Supplementary material for: Valvular and ascending aortic hemodynamics of the On-X aortic valved conduit by same-day echocardiography and 4D flow MRI
Source: Front Cardiovasc Med. 2023 Nov 14;10:1256420. doi: 10.3389/fcvm.2023.1256420 (PMC10682731; doi:10.3389/fcvm.2023.1256420)
Supplement: Supplementary file 7 [file Datasheet1.docx]

Supplementary Material

# Description of viscous energy loss rate

Viscous energy loss rate (VELR) represents the rate of flow mechanical energy loss due to friction between two adjacent fluid layers moving at a different velocity (i.e., fluid shear).[1] Assuming blood as an incompressible and Newtonian fluid, VELR per unit volume can be calculated as,

|  | $VELR=\frac{1}{2}\mu\sum_{i=1}^{3} \sum_{j=1}^{3} \left( \frac{\partial u_{i}}{\partial x_{j}}+\frac{\partial u_{j}}{\partial x_{i}} \right)^{2}-\frac{2}{3} \mu\sum_{i=1}^{3} \left( \frac{\partial u_{i}}{\partial x_{i}} \right)^{2}$ |  |
| --- | --- | --- |

where $\mu$ indicates the dynamic viscosity and $u_{i}$ indicates the velocity component along $x_{i}$ direction where $x_{1}$, $x_{2}$ and $x_{3}$ correspond to the Cartesian axes $x$, $y$ and $z$, respectively. A dynamic viscosity of 3.2 cP was assumed for blood in the aorta.[2]

# Description of vorticity

Vorticity represents the angular velocity vector of a fluid element under rotation present when flow becomes spatially nonuniform and increases as flow exhibits stronger turbulent or faster vortical flow motion. Vorticity ($\boldsymbol{\omega})$ is a vector quantity calculated by taking the curl to the velocity vectors,

|  | $\boldsymbol{\omega}=\left[ \begin{matrix} \frac{\partial u_{3}}{\partial x_{2}}-\frac{\partial u_{2}}{\partial x_{3}}, & \frac{\partial u_{1}}{\partial x_{3}}-\frac{\partial u_{3}}{\partial x_{1}}, & \frac{\partial u_{2}}{\partial x_{1}}-\frac{\partial u_{1}}{\partial x_{2}} \end{matrix} \right]$ |  |  |
| --- | --- | --- | --- |

where first, second and third element corresponds to the vorticity components along $x_{1}$, $x_{2}$ and $x_{3}$ axis, respectively.

1. P. K. Kundu and I. M. Cohen. Fluid Mechanics, fourth edition. Elsevier; 2008. p. 112-113.

2. N. Westerhof, N. Stergiopulos, M. I. Noble, et al. Snapshots of hemodynamics: an aid for clinical research and graduate education: Springer; 2018.
